# Association between levels of IgG antibodies from vaccines and Omicron symptomatic infection among children and adolescents in China

**DOI:** 10.3389/fmed.2023.1240340

**Published:** 2023-08-29

**Authors:** Xinying Chen, Junbin Hong, Lijun Deng, Heng Weng, Teng Huang, Li Wang, Aihua Ou, Yuxia Li, Bo Yu, Jianwen Guo, Jinghua Yang

**Affiliations:** ^1^Department of Pediatrics, The Second Affiliated Hospital of Guangzhou University of Chinese Medicine, Guangzhou, China; ^2^Xiaorong Luo’s Renowned Expert Inheritance Studio, Guangdong Provincial Hospital of Chinese Medicine, Guangzhou, China; ^3^The Second Clinical Medical College, Guangzhou University of Chinese Medicine, Guangzhou, China; ^4^State Key Laboratory of Dampness Syndrome of Chinese Medicine, The Second Affiliated Hospital of Guangzhou University of Chinese Medicine, Guangzhou, China; ^5^Department of Lab, The No.2 People's Hospital of Lanzhou, Lanzhou, China; ^6^Department of Pediatrics, The Affiliated Hospital of Gansu University of Chinese Medicine, Lanzhou, China; ^7^Department of Surgery, The No.2 People's Hospital of Lanzhou, Lanzhou, China; ^8^Department of Neurology, The Second Affiliated Hospital of Guangzhou University of Chinese Medicine, Guangzhou, China

**Keywords:** IgG levels, antibody, SARS-CoV-2, Omicron, symptomatic infection

## Abstract

**Background:**

Measurements of IgG antibodies to wild-type SARS-CoV-2 antigens can assess vaccine efficacy, but the absolute risk of Omicron symptomatic infection at different IgG levels for children and adolescents remains uncertain, as well as the minimum effective antibody level. We sought to determine the relationship between the tertiles of IgG antibodies to wild-type SARS-CoV-2 antigens and children with symptomatic infection of the pandemic and duration to negative conversion in China for the first time.

**Methods:**

A retrospective study was conducted, including 168 participants under 18 years old from the No.2 People’s Hospital of Lanzhou, China, diagnosed with Omicron variant BA.2.38 between July 8, 2022, and August 2, 2022. We calculated odds ratios (OR) in univariate and multivariate regression to assess the association of symptomatic infection with the tertiles of IgG, respectively. Kaplan–Meier curves and Cox proportional hazards regression were used to evaluate the relationship between IgG level and negative conversion time.

**Results:**

The average age of the 168 children included in this study was 7.2 (4.7) years old, 133 (79.2%) were symptomatic patients, and the average negative conversion time was 12.2 (3.5) days. The participants with high IgG levels were less likely to become symptomatic, had a shorter turnaround time, and had higher values of IgM and nucleic acid CT. Compared to those with the lowest tertile of IgG, patients with the highest tertile had a 91% lower risk of developing a symptomatic infection after fully adjusting for confounders (OR = 0.09, 95% CI, 0.02-0.36, *p* = 0.001). There’s no robust relationship between IgG level and negative conversion time in multivariate Cox regression.

**Conclusion:**

The risk of developing a symptomatic infection can be predicted independently by tertiles of IgG antibodies to wild-type SARS-CoV-2 antigens. High IgG levels can inhibit viral replication, vastly reduce the risk of symptomatic infections and promote a virus-negative conversion, especially when IgG quantitative detection was ≥3.44 S/CO, a potential threshold for protection and booster strategy in the future. More data and research are needed in the future to validate the predictive models.

## 1. Introduction

The emergence of the Omicron variant of severe acute respiratory syndrome coronavirus 2 (SARS-CoV-2), which has led to an increase in the number of global infections in a short period, is highly transmissible and has caused widespread concern (1, 2). It has become a major variant in many countries around the world, and China is no exception (3, 4). Also, it is associated with increased infection rates and higher hospitalization rates in children (5, 6). More importantly, the Omicron variant and its multiple sub-lineages can undergo immune escape, which not only increases resistance in convalescent plasma but also decreases vaccine efficacy (7, 8). Recent studies have shown that vaccine boosters can help prevent Novel Coronavirus (2019-nCoV) infection in children and reduce the risk of severe disease (9, 10). However, boosters are a key part to maintain high neutralizing antibody levels, which carries certain risks and complications, such as myocarditis, pericarditis, thrombosis and so on (11). Thus, it is necessary to seek improved methods and optimal recommendations on booster decision-making.

Studies have shown that antibody levels, especially neutralizing antibody levels, are highly predictive of vaccine efficacy, and neutralizing antibody levels are highly predictive of immune protection against symptomatic or severe SARS-CoV-2 infection (12, 13). Recently, it has been confirmed that anti-spike SARS-CoV-2 antibodies help reduce the risk of breakthrough infections among adults (14). Moreover, immunoglobulin G (IgG) levels of SARS-CoV-2-specific antibodies have been confirmed to be effective in predicting neutralizing antibody levels and assessing vaccine efficacy (15). In China, tests for IgG antibodies against SARS-CoV-2, detected by chemiluminescence, a semi-quantitative detection method, are more readily available than those for the neutralizing antibody (16, 17). However, studies on the association between the levels of antibodies against wild-type SARS-CoV-2 antigens induced by inactivated vaccines among Chinese children and the risk prediction of time to negative conversion are still rare. Besides, the accurate quality and quantity of neutralizing antibodies required to prevent humans from infection with SARS-CoV-2 remain uncertain (18).

Since vaccine resources are limited, it is critical to optimize booster vaccinations based on antibody levels. Here, we describe the relationship between IgG levels, other available laboratory markers, symptomatic infections, and time to negative conversion. We sought to examine the hypothesis that tertiles of IgG might identify populations at increased risk of symptomatic infections and that it takes longer for those children with a low IgG level to turn negative.

## 2. Materials and methods

### 2.1. Study design and population

This retrospective cohort study was conducted from August 20, 2022, to September 2, 2022, using the clinical medical records in the No.2 People’s Hospital of Lanzhou between July 8, 2022, and August 2, 2022. All patients under 18 years old with Omicron variant BA.2.38 were included in the study if they had the laboratory tests within 24 h after admission, particularly IgG against SARS-CoV-2. Those were excluded if their findings of IgG were unavailable or available after the seventh day of disease progression calculated from the first positive nucleic acid test. Neither the patient nor the patient’s family members denied any history of the previous infection. This study was reviewed and approved by the Ethics Committee of Guangdong Provincial Hospital of Chinese Medicine (Ethic number: ZF2022-246-01), and informed consent was obtained from the guardians of the participants. Additionally, the Reporting Guidelines for Strengthening the Reporting of Observational Studies in Epidemiology (STROBE) were followed.

**Figure 1:**
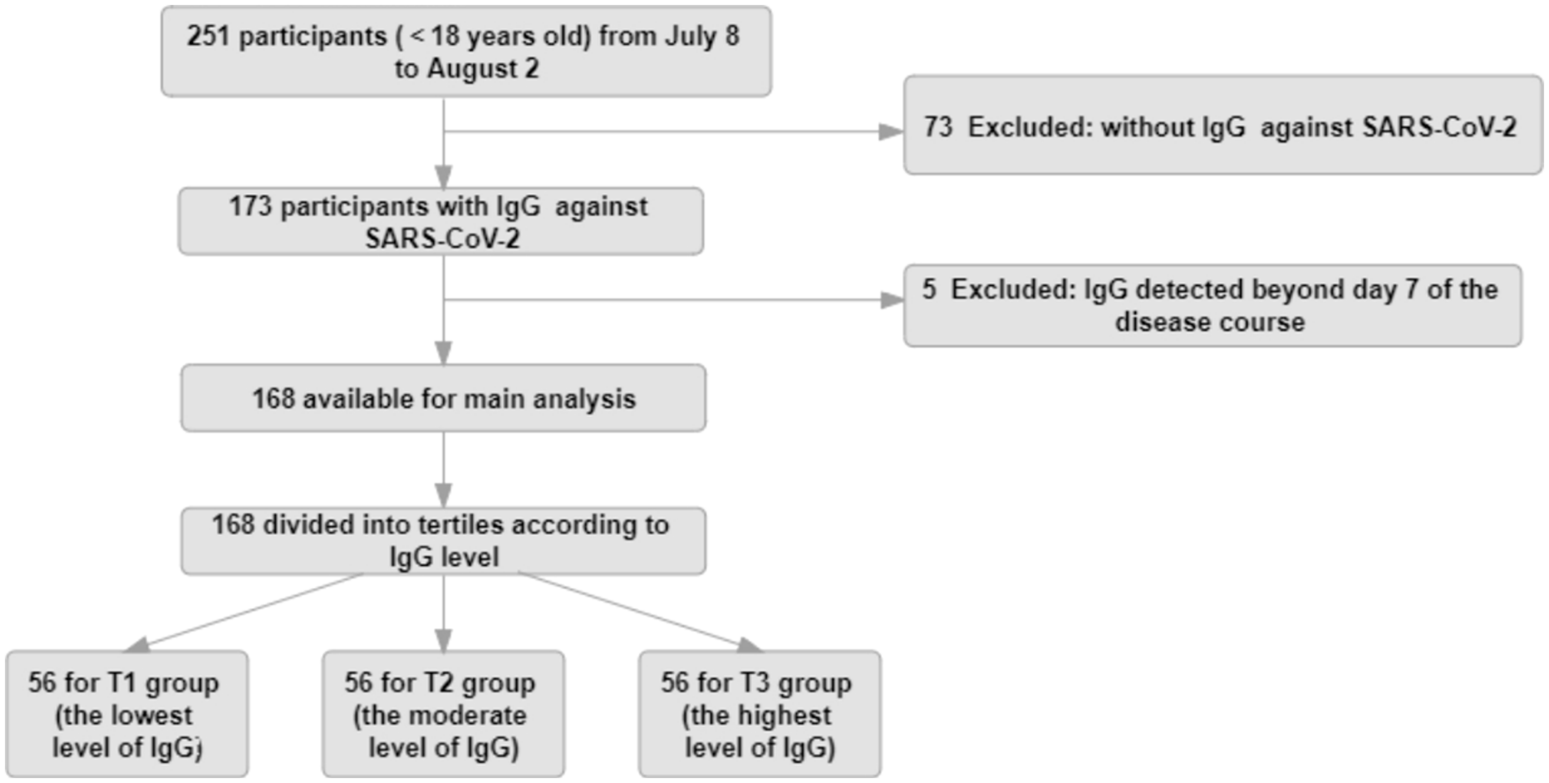
Study flow diagram. The study population for the main analysis (*n* = 168) consisted of participants continuously enrolled in the No.2 People’s Hospital of Lanzhou, who had complete data, such as IgG values within 7 days during the course of disease, symptomatic infection, and negative conversion time.

All patients with enough identifying information were able to be associated with symptomatic infection and time to negative conversion. Data from 168 patients were computed in the analysis after the study exclusion criteria were applied (Figure 1).

### 2.2. Study variables

By retrieving the electronic medical record system, the data collected embraced demographic information, clinical signs and symptoms, vaccinations, comorbidities (nephrotic syndrome, hemopathy, other condition with immune deficiency or suppression and etc.), and laboratory values, such as blood counts (leukocytes, neutrophils, lymphocytes, hemoglobin, platelet count), inflammatory markers (C-reactive protein [CRP], procalcitonin [PCT], serum amyloid [SAA], interleukin-6 [IL-6]), liver function parameters (alanine aminotransferase [ALT], aspartate aminotransferase [AST]), myocardial enzymes (creatine kinase [CK], CK isoenzyme MB [CK-MB], lactate dehydrogenase [LDH]), coagulation function indicators (D-dimer [DDi], fibrinogen [FIB]), cycle threshold (CT) of SARS-CoV-2 nucleic acid test (CT of nucleocapsid protein [N-CT], CT of open reading frame 1ab [ORF1ab-CT]), and antibodies against 2019-nCoV (immunoglobulin G [IgG], immunoglobulin M [IgM]), which were indirectly detected with wild-type SARS-CoV-2 recombinant antigens including the nucleoprotein and a peptide from the spike protein in an available Magnetic Chemiluminescence Enzyme Immunoassay Kit of Bioscience. Add reagents, samples, and magnetic beads successively, and mix to react thoroughly. Then wash, and add pre-excitation and excitation solutions to detect the signal as a relative light unit (RLU), which is further converted into the final titer reading, expressed in the titer unit of S/CO (16, 19).

Asymptomatic infection was defined as the absence of clinical symptoms and positive signs on physical examination from the time of diagnosis of Omicron infection to the end of hospitalization, while symptomatic infection was defined as the presence of any clinical symptom reported by the children or their guardians or positive sign retrieved from electronic medical record system (20). Besides, children with peripheral capillary oxygen saturation < 93% were diagnosed with severe infection (20). Time to negative conversion, also called turnaround time, was described as the duration from the date of the first nucleic acid positive to the first date of two consecutive nucleic acid negatives.

### 2.3. Outcomes

The outcome indicators of this study included the occurrence of symptomatic infection and the negative conversion time.

### 2.4. Statistical analysis

Descriptive analyses demonstrate distributions of demographic, clinical, and laboratory characteristics at baseline concerning tertiles of IgG levels (the first tertile of IgG, T1, serves as a reference; the third tertile, T3, represents the highest levels). For numerical variables, we used the mean (standard deviation [SD]) or median (interquartile range [IQR]), and for categorical variables, we used the frequency (%). Binary logistic regression models in univariate and multivariate analyses were used to determine whether IgG level is a significant independent predictor of symptomatic infection, with odds ratios (OR) and 95% confidence intervals (CI), respectively. Also, we assessed the relationship between IgG levels and time to negative conversion with Kaplan–Meier (KM) survival curves and the Breslow test. Hazard ratios (HR) and 95% CI were regulated using Cox proportional hazards regression models to analyze the risk of negative conversion associated with IgG levels. In the multivariate regression analysis, candidate variables with a *p*-value < 0.20 in univariate analysis and those thought to be clinically relevant were included in unconditional stepwise logistic regression analysis using the maximum likelihood estimation method (21, 22). For each model, the lowest value was the reference. It was statistically significant in 2-sided tests with a *p*-value < 0.05. All statistics were conducted in SPSS software version 25.0, except KM survival curves using the R language and the forest plot in MedCalc software.

## 3. Results

### 3.1. Characteristics of participants with different IgG levels

Among the 168 participants, they were healthy and had no history of SARS-CoV-2 infection previously; the mean age at baseline was 7.2 (4.7) years; 89 (53.0%) were male; 133 (79.2%) were symptomatic; 2 (1.2) were diagnosed with severe infection without respiratory failure, septic shock, and/or multiple organ dysfunction (one in T1 and the other in T3); and the mean turnaround time was 12.2 (3.5) days. The study population featured according to the tertiles of IgG against 2019-nCoV (Table 1). T1 tertile of IgG was 0.01-0.42 S/CO; T2 of that was 0.43-3.43 S/CO; T3 of that was 3.44-134.32 S/CO. The mean time from the first positive virus PCR to antibody detection was 1.7 (1.6) days with 1.7 (1.5) days in the T1 group, 1.4 (1.1) days in the T2 group, and 1.8 (2.0) days in the T3 group respectively, and there was no significant difference between IgG groups. As for the vaccines, the children in China could be vaccinated with inactivated vaccines only and 110 individuals had a history of vaccination. Briefly, the mean time from the first positive virus PCR to antibody detection and the type of vaccines did not make sense to the level of IgG.

**Table 1:** Patient characteristics according to tertiles of IgG^a1^.

Variables		All patients (*N* = 168)	Tertiles of SARS-CoV-2-Ig G		
			T1:0.01–0.42 S/CO (*n* = 56)	T2:0.43–3.43 S/CO (*n* = 56)	T3:3.44–134.32 S/CO (*n* = 56)
Gender	Male	89 (53.0)	31 (55.4)	32 (57.1)	26 (46.4)
	Female	79 (47.0)	25 (44.6)	24 (42.9)	30 (53.6)
Age group, y	0-6	78 (46.4)	47 (83.9)	10 (17.9)	21 (37.5)
	>6, ≤12	61 (36.3)	4 (7.2)	34 (60.7)	23 (41.1)
	>12, <18	29 (17.3)	5 (8.9)	12 (21.4)	12 (21.4)
Time from the first positive virus PCR to antibody detection	Mean (SD), d	1.7 (1.6)	1.7 (1.5)	1.4 (1.1)	1.8 (2.0)
Severity at the end of observation	Asymptomatic	35 (20.8)	4 (7.1)	10 (17.9)	21 (37.5)
	Symptomatic	133 (79.2)	52 (92.9)	46 (82.1)	35 (62.5)
Severe infection	Yes	2 (1.2)	1 (1.8)	0 (0)	1 (1.8)
	No	164 (98.8)	55 (98.2)	56 (100)	55 (98.2)
Vaccination	Unvaccinated	50 (29.7)	45 (80.4)	5 (8.9)	0 (0.0)
	1 dose	3 (1.8)	0 (0.0)	2 (3.6)	1 (1.8)
	2 doses	95 (56.5)	4 (7.1)	44 (78.6)	47 (83.9)
	3 doses	5 (3.0)	1 (1.8)	3 (5.3)	1 (1.8)
	Vaccinated with uncertain dose	7 (4.2)	0 (0.0)	0 (0.0)	7 (12.5)
	Missing	8 (4.8)	6 (10.7)	2 (3.6)	0 (0.0)
Negative conversion time	Mean (SD), d	12.2 (3.5)	12.8 (3.5)	12.6 (3.5)	11.2 (3.3)
	≤8 days	19 (11.3)	3 (5.4)	2 (3.6)	14 (25.0)
	≤9 days	35 (19.0)	8 (14.3)	7 (12.5)	17 (30.4)
	≤10 days	62 (36.9)	19 (33.9)	15 (26.8)	28 (50.0)
Symptoms and signs					
Fever		89 (53.0)	43 (76.8)	25 (44.6)	21 (37.5)
Stages of fever	No.	161	53	55	53
	<37.3°C	79 (49.1)	13 (24.5)	31 (56.4)	35 (66.0)
	37.3-39°C	52 (32.3)	23 (43.4)	17 (30.9)	12 (22.7)
	≥39.1°C	30 (18.6)	17 (32.1)	7 (12.7)	6 (11.3)
Cough		77 (45.8)	23 (41.1)	30 (53.6)	24 (42.9)
Sputum		37 (22.0)	13 (23.2)	14 (25.0)	10 (17.9)
Stuffy nose		12 (7.1)	5 (8.9)	3 (5.4)	4 (7.1)
Runny nose		18 (10.7)	7 (12.5)	6 (10.7)	5 (8.9)
Fatigue		15 (8.9)	7 (12.5)	4 (7.1)	4 (7.1)
Excessive sweating		1 (0.6)	0 (0.0)	0 (0.0)	1 (1.8)
Dry throat		3 (1.8)	2 (3.6)	0 (0.0)	1 (1.8)
Sore throat		15 (8.9)	1 (1.8)	12 (21.4)	2 (3.6)
Tickle in throat		10 (6.0)	1 (1.8)	7 (12.5)	2 (3.6)
Headache		9 (5.4)	3 (5.4)	4 (7.1)	2 (3.6)
Body pain		1 (0.6)	1 (1.8)	0 (0.0)	0 (0.0)
Lost sense of smell		1 (0.6)	1 (1.8)	0 (0.0)	0 (0.0)
Abdominal pain		6 (3.6)	3 (5.4)	1 (1.8)	2 (3.6)
Abdominal distension		2 (1.2)	2 (3.6)	0 (0.0)	0 (0.0)
Nausea		8 (4.8)	2 (3.6)	4 (7.1)	2 (3.6)
Vomiting		7 (4.2)	3 (5.4)	3 (5.4)	1 (1.8)
Decreased appetite		30 (17.9)	18 (32.1)	7 (12.5)	5 (8.9)
Bad sleep		15 (8.9)	12 (21.4)	2 (3.6)	1 (1.8)
Abnormal bowel movements		18 (10.7)	9 (16.1)	7 (12.5)	2 (3.6)
Irritable		22 (13.1)	17 (30.4)	2 (3.6)	3 (5.4)
Conjunctival injection		1 (0.6)	0 (0.0)	0 (0.0)	1 (1.8)
SpO2, %	<93	2 (1.2)	1 (1.8)	0 (0.0)	1 (1.8)
	93–94	2 (1.2)	1 (1.8)	0 (0.0)	1 (1.8)
	≥95	164 (97.6)	54 (96.4)	56 (100.0)	54 (96.4)
Laboratory findings					
WBC	No.	167	55	56	56
	mean (SD),10^9/L	6.1 (2.4)	7.0 (3.0)	5.1 (1.7)	6.1 (2.0)
NEUT	No.	167	55	56	56
	mean (SD),10^9/L	3.0 (1.7)	2.9 (1.9)	2.8 (1.5)	3.3 (1.6)
LYM	No.	167	55	56	56
	mean (SD),10^9/L	2.4 (1.8)	3.4 (2.4)	1.8 (1.3)	2.1 (1.0)
Hb	No.	167	55	56	56
	mean (SD), g/L	131.1 (11.5)	125.4 (12.2)	135.0 (11.3)	132.7 (8.8)
PLT	No.	167	55	56	56
	mean (SD),10^9/L	238.5 (70.7)	253.4 (82.7)	222.7 (52.4)	239.7 (71.7)
CRP levels	No.	165	53	56	56
	≤10 mg/L	151 (91.5)	52 (98.1)	47 (83.9)	52 (92.9)
	11-19 mg/L	7 (4.2)	1 (1.9)	6 (10.7)	0 (0.0)
	≥20 mg/L	7 (4.2)	0 (0.0)	3 (5.4)	4 (7.1)
PCT levels	No.	155	52	53	50
	<0.1 ng/ml	105 (67.7)	28 (53.8)	43 (81.1)	34 (68)
	0.1-0.25 ng/ml	30 (19.4)	15 (28.8)	6 (11.3)	9 (18)
	>0.25 ng/ml	20 (12.9)	9 (17.3)	4 (7.5)	7 (14)
SAA levels	No.	156	54	52	50
	≤10 μg/ml	2 (1.3)	0 (0.0)	0 (0.0)	2 (4.0)
	>10 μg/ml	154 (98.7)	54 (100.0)	52 (100.0)	48 (96.0)
IL-6 levels	No.	156	52	53	51
	≤7 pg./ml	121 (77.6)	37 (71.2)	44 (83)	40 (78.4)
	>7 pg./ml	35 (22.4)	15 (28.8)	9 (17)	11 (21.6)
ALT levels	No.	167	56	56	55
	≤40 U/L	149 (89.2)	47 (83.9)	50 (89.3)	52 (94.5)
	41–59 U/L	11 (6.6)	4 (7.2)	5 (8.9)	2 (3.6)
	≥60 U/L	7 (4.2)	5 (8.9)	1 (1.8)	1 (1.8)
AST levels	No.	168	56	56	56
	≤40 U/L	132 (78.6)	27 (48.2)	51 (91.1)	55 (96.4)
	41–59 U/L	21 (12.5)	16 (28.6)	4 (7.1)	1 (1.8)
	≥60 U/L	15 (8.9)	13 (23.2)	1 (1.8)	1 (1.8)
CK	No.	166	55	56	55
	Mean (SD), U/L	98.0 (41.8)	111.3 (43.9)	98.9 (42.2)	83.9 (34.8)
CK-MB	No.	166	55	56	55
	Mean (SD), U/L	19.6 (8.9)	25.2 (9.5)	18.1 (9.0)	15.6 (4.1)
LDH	No.	167	55	56	56
	Mean (SD), U/L	258.0 (67.5)	304.1 (68.6)	238.5 (61.0)	232.3 (47.0)
DDi levels	No.	158	56	50	52
	≤0.50 μg/ml	121 (76.6)	37 (66.1)	39 (78)	45 (86.5)
	0.51–0.99 μg/ml	26 (16.5)	13 (23.2)	7 (14.0)	6 (11.5)
	≥1.00 μg/ml	11 (7.0)	6 (10.7)	4 (8.0)	1 (1.9)
FIB	No.	163	56	52	55
	Mean (SD), g/L	2.3 (0.6)	2.0 (0.5)	2.5 (0.5)	2.4 (0.5)
N-CT	No.	155	55	52	48
	Mean (SD)	27.6 (4.8)	25.2 (4.2)	27.8 (4.9)	30.2 (4.1)
ORF1ab-CT	No.	151	57	52	44
	Mean (SD)	27.3 (5.4)	24.6 (4.5)	27.8 (5.4)	30.2 (5.0)
SARS-CoV-2 IgM	No.	168	56	56	56
	Median (IQR), S/CO	0.14 (0.07,0.30)	0.08 (0.05,0.17)	0.15 (0.07,0.33)	0.22 (0.13,0.43)

WBC, white blood cell count (5.0–12.0) × 10^9/L; NEUT, neutrophil count (2.0-7.0) × 10^9/L; LYM, lymphocyte count (0.8-4.0) × 10^9/L; Hb, hemoglobin; PLT, platelet count (100-300) × 10^9/L; CRP, C-reactive protein; PCT, procalcitonin (0.000–0.046) ng/l; SAA, serum amyloid (0.00-10.00) μg/l; IL- 6, interleukin 6 (0.00-10.00) pg/l; ALT, alanine aminotransferase (0–50) U/L; AST, aspartate aminotransferase (0–40) U/L; CK, creatine kinase (24–194) U/L; CK-MB, creatine kinase isoenzyme MB (0–25) U/L; LDH, lactate dehydrogenase (109–245) U/L; DDi, D-dimer (0.00-0.55) μg/ml; FIB, fibrinogen (2.00–4.00) μg/ml; N-CT, cycle threshold of nucleocapsid protein < 40; ORF1ab-CT, cycle threshold of open reading frame 1ab, < 40; No., number of valid cases; a1, the data are presented as number (percent) unless otherwise indicated.

Compared to those with the lowest level of IgG (T1), participants with the highest level of IgG (T3) were more likely to complete two or three doses of vaccination (48 [85.7%] vs. 5 [8.9%]); less likely to develop a symptomatic infection (35 [62.5%] vs. 51 [91.1%]); less likely to have a fever (21 [37.5%] vs. 43 [76.8%]), decreased appetite(5 [8.9%] vs. 30 [17.9%]), bad sleep (12[21.4%] vs. 1[1.8%]), elevated AST (>40 U/L) (2 [3.6%] vs. 29 [51.8%]), elevated DDi (7 [13.4%] vs. 19 [33.9%]), lower level of CK (mean[SD] 83.9 [34.8]) vs. (111.3[43.9]), lower level of CK-MB (mean[SD] 15.6[4.1] vs. 25.2[9.5]), and lower level of LDH (mean[SD], 232.3[47.0] vs. 304.1[68.6]), higher value of N-CT (mean[SD], 30.2[4.1]vs. 25.2[4.2]), higher value of ORF1ab-CT (mean[SD], 30.2[5.0] vs. 24.6[4.5]), and higher value of IgM (median[IQR], 0.22[0.13–0.43] vs. 0.08[0.05, 0.17]); more likely to need shorter time to negative conversion (mean[SD] days, 11.2 [3.3] vs. 12.8 [3.5], respectively); more likely to have a negative conversion of 8 days, 9 days and 10 days after diagnosis of infection (14 [25.0%] vs. 3 [5.4%]; 17 [30.4%] vs. 8 [14.3%]; 28 [50.0%] vs. 19 [33.9%]).

### 3.2. Risk of symptomatic infection associated with IgG levels and clinical risk factors

Table 2 shows univariate and multivariate regression models assessing baseline variables and occurrence of symptomatic infection.

**Table 2:** Binary regression of symptomatic infection.

Variables		Asymptomatic (*n* = 35)	Symptomatic (*n* = 133)	Model 1		Model 2		Model 3	
				Univariable OR (95%CI)	*P*	Multivariable OR (95%CI)	*P*	Multivariable OR (95%CI)	*P*
Gender	Female	15 (42.9)	64 (48.1)	1.00 (Ref.)					
	Male	20 (57.1)	69 (51.9)	1.24 (0.58–2.62)	0.579	NA	NA	NA	NA
Age group, y	0-6	14 (40.0)	64 (48.1)	1.00(Ref.)					
	>6, ≤12	13 (37.1)	48 (36.1)	0.81 (0.35–1.88)	0.619	NA	NA	NA	NA
	>12, <18	8 (22.9)	21 (15.8)	0.57 (0.21–1.56)	0.276	NA	NA	NA	NA
Vaccination^b1^	Unvaccinated	5 (14.3)	45 (36.0)	1.00(Ref.)					
	Vaccinated	30 (85.7)	80 (64.0)	0.30 (0.11–0.82)	0.019	NA	NA	NA	NA
Tertiles of IgG	T1(0.01–0.42 S/CO)	4 (11.4)	52 (39.1)	1.00(Ref.)		1.00(Ref.)		1.00(Ref.)	
	T2(0.43–3.43 S/CO)	10 (28.6)	46 (34.6)	0.35 (0.10–1.21)	0.097	0.19 (0.04-0.82)	0.026	0.19 (0.04-0.83)	0.028
	T3(3.44–134.32 S/CO)	21 (60.0)	35 (26.3)	0.13 (0.04-0.41)	<0.001	0.09 (0.02-0.35)	0.001	0.09 (0.02-0.36)	0.001
CRP levels	No.	35	130						
	≤10 mg/L	34 (97.1)	117 (90.0)	1.00(Ref.)					
	11–19 mg/L	0 (0.0)	7 (5.4)	469454227.8 (0.00 − +∞)	0.999	NA	NA	NA	NA
	≥20 mg/L	1 (2.9)	6 (4.6)	1.74 (0.20–14.99)	0.612	NA	NA	NA	NA
PCT levels	No.	30	125						
	<0.1 ng/ml	24 (80.0)	81 (64.8)	1.00(Ref.)					
	0.1–0.25 ng/ml	4 (13.3)	26 (20.8)	1.93 (0.61–6.06)	0.263	NA	NA	NA	NA
	>0.25 ng/ml	2 (6.7)	18 (14.4)	2.67 (0.58–12.32)	0.209	NA	NA	NA	NA
SAA levels	No.	29	127						
	≤10 μg/ml	1 (3.4)	1 (0.8)	1.00(Ref.)					
	>10 μg/ml	28 (96.6)	126 (99.2)	4.50 (0.27–74.14)	0.293	NA	NA	NA	NA
IL-6 levels	No.	31	125						
	≤7 pg./ml	26 (83.9)	95 (76.0)	1.00(Ref.)					
	>7 pg./ml	5 (16.1)	30 (24.0)	1.64 (0.58–4.65)	0.351	NA	NA	NA	NA
ALT levels	No.	35	132						
	≤40 U/L	30 (85.7)	119 (90.2)	1.00(Ref.)					
	41-59 U/L	3 (8.6)	8 (6.1)	0.67 (0.17–2.69)	0.574	NA	NA	NA	NA
	≥60 U/L	2 (5.7)	5 (3.8)	0.63 (0.12–3.41)	0.592	NA	NA	NA	NA
AST levels	No.	35	133						
	≤40 U/L	30 (85.7)	102 (76.7)	1.00(Ref.)					
	41–59 U/L	2 (5.7)	19 (14.3)	2.80 (0.62–12.68)	0.183	NA	NA	NA	NA
	≥60 U/L	3 (8.6)	12 (9.0)	1.18 (0.31–4.44)	0.811	NA	NA	NA	NA
DDi levels	No.	34	124						
	≤0.50 μg/ml	27 (79.4)	94 (75.8)	1.00(Ref.)					
	0.51–0.99 μg/ml	6 (17.6)	20 (16.1)	0.96 (0.35–2.62)	0.933	NA	NA	NA	NA
	≥1.00 μg/ml	1 (2.9)	10 (8.1)	2.87 (0.35–23.45)	0.325	NA	NA	NA	NA
WBC	No.	35	132						
	Mean (SD), 10^9/L	6.1 (1.9)	6.0 (2.5)	0.99 (0.85–1.16)	0.939	NA	NA	NA	NA
NEUT	No.	35	132						
	Mean (SD), 10^9/L	2.7 (1.3)	3.1 (1.7)	1.15 (0.89–1.48)	0.274	NA	NA	NA	NA
LYM	No.	35	132						
	Mean (SD), 10^9/L	2.7 (1.6)	2.4 (1.9)	0.90 (0.75–1.09)	0.294	NA	NA	NA	NA
Hb	No.	35	132						
	Mean (SD), g/L	129.6 (9.2)	131.4 (12.1)	1.01 (0.98–1.05)	0.401	NA	NA	NA	NA
PLT	No.	35	132						
	Mean (SD), 10^9/L	247.1 (72.2)	236.2 (70.4)	1.00 (0.99–1.00)	0.418	NA	NA	NA	NA
FIB	No.	35	128						
	Mean (SD), g/L	2.2 (0.4)	2.4 (0.6)	1.79 (0.89–3.60)	0.101	4.18 (1.50–11.63)	0.006	4.15 (1.49–11.59)	0.007
N-CT	No.	28	127						
	Mean (SD)	29.1 (4.4)	27.3 (4.9)	0.92 (0.84–1.01)	0.068	NA	NA	NA	NA
ORF1ab-CT	No.	26	125						
	Mean (SD)	28.9 (5.4)	27.0 (5.4)	0.93 (0.86–1.01)	0.096	NA	NA	NA	NA
CK	No.	35	131						
	Mean (SD), U/L	84.0 (34.9)	101.8 (42.7)	1.01 (1.00–1.02)	0.027	NA	NA	NA	NA
CK-MB	No.	35	131						
	Mean (SD), U/L	17.2 (6.0)	20.3 (9.4)	1.05 (1.00–1.11)	0.070	NA	NA	NA	NA
LDH	No.	35	132						
	Mean (SD), U/L	231.7 (46.7)	264.9 (70.5)	1.01 (1.00–1.02)	0.010	NA	NA	NA	NA
SARS-CoV-2 IgM	No.	35	133						
	Median (IQR), S/CO	0.2 (0.1, 0.3)	0.1 (0.1, 0.3)	0.79 (0.44–1.42)	0.427	NA	NA	NA	NA

The Spearman correlation coefficient between SARS-CoV-2 IgG antibody level as a continuous variable and vaccine dose was 0.711 (*P* < 0.001), and that between IgG tertiles and vaccine dose was 0.719 (*P* < 0.001), so we believe that IgG tertiles can reflect the vaccination status. Furthermore, in univariate regression analysis, the relationship between IgG tertiles and symptomatic infection was more robust than that between vaccine dose and symptomatic infection. Model 2, multivariate regression of IgG tertiles and occurrence of symptomatic infection adjusted for age, gender, vaccination, FIB, N-CT, ORF1ab-CT, CK, CK-MB, and LDH. Model 3, multivariate regression of IgG tertiles and occurrence of symptomatic infection adjusted for age, gender, vaccination, LYM, FIB, N-CT, ORF1ab-CT, CK, CK-MB, LDH. NA, not applicable. b1, the vaccine status of the 8 patients was missing. Missing data were not included in both univariate and multivariate regression.

**Figure 2:**
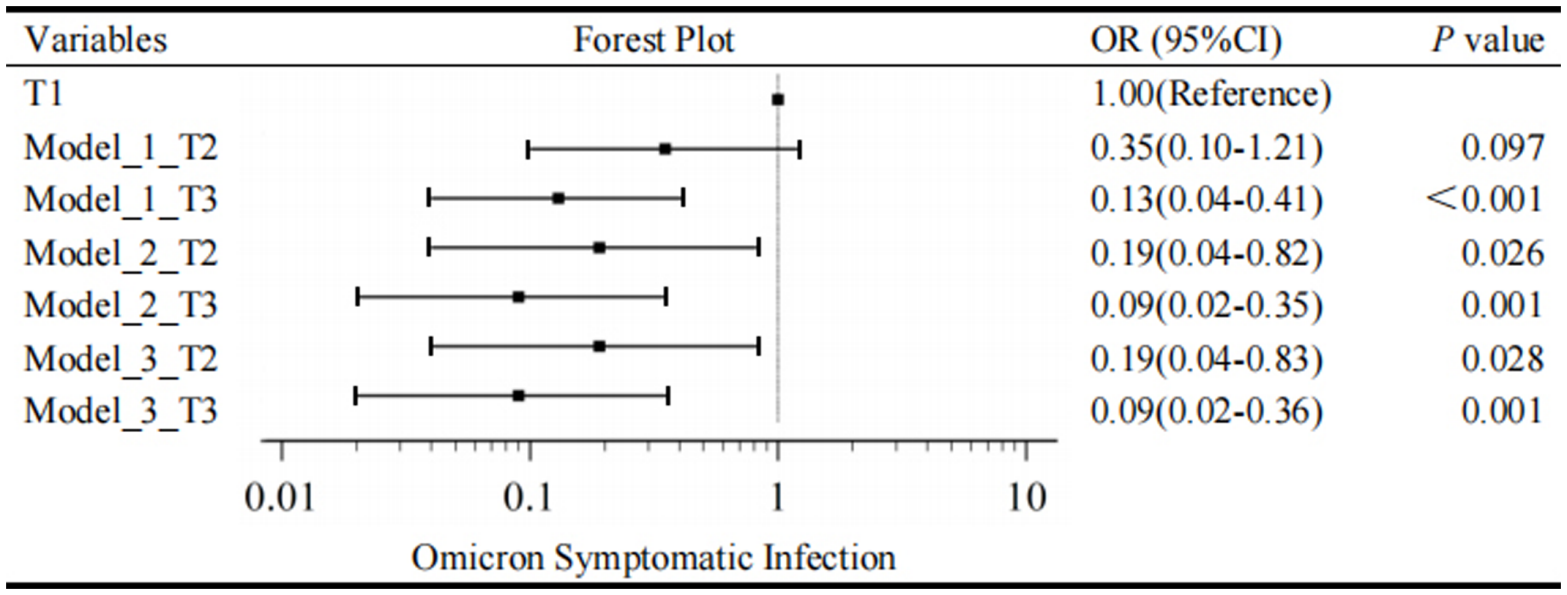
Forest plot for Omicron symptomatic infection by tertiles of IgG. T1, the first tertile of IgG (lowest), as the reference in Model 1-3. The outcome was the presence of Omicron Symptomatic Infection.

Firstly, the univariate regression model indicated the variables, such as gender, age group, CRP levels, PCT levels, SAA levels, IL-6 levels, ALT levels, DDi levels, WBC, NEUT, LYM, Hb, PLT, and SARS-CoV-2 IgM, made no statistic difference between asymptomatic and symptomatic infection with a *p*-value ≥0.20. Children vaccinated before were less likely to be symptomatic than those unvaccinated (OR = 0.30, 95% CI, 0.11–0.82, *p* = 0.019). Unadjusted regression analysis demonstrated a decrease in risk of symptomatic infection in the elevated tertile of IgG with a significantly decreasing OR in Model 1. The T3 group of IgG had an 87% lower risk of developing a symptomatic infection compared with T1 group (OR = 0.13, 95% CI, 0.04–0.41, *p* < 0.001); the T2 group of IgG had an 65% lower risk of developing a symptomatic infection compared to T1 group (OR = 0.35, 95% CI, 0.10–1.21, *p* < 0.097) (Figure 2). LDH was also positively associated with symptomatic infection (OR = 1.01, 95% CI, 1.00–1.02, *p* = 0.010). Besides, univariate analysis screened out candidate variables with a *p*-value <0.20, such as N-CT (OR = 0.92, 95% CI, 0.84–1.01, *p* = 0.068), ORF1ab-CT (OR = 0.93, 95% CI, 0.86–1.01, *p* = 0.096), CK (OR = 1.01, 95% CI, 1.00–1.02, *p* = 0.027), CK-MB (OR = 1.05, 95% CI, 1.00–1.11, *p* = 0.070), and FIB (OR = 1.79, 95% CI, 0.89–3.60, *p* = 0.101).

Secondly, a multivariate regression model was conducted to evaluate the association between IgG tertiles and the occurrence of symptomatic infection. As mentioned above, IgG tertiles, vaccination, FIB, N-CT, ORF1ab-CT, CK, CK-MB, and LDH were included in unconditional stepwise logistic regression analysis. As for age and gender, they were the most common confounders in the clinical study, so they were also included in the adjusted model. After adjusted for these confounding factors, Model 2 showed that the T3 group of IgG had a 91% lower risk of developing a symptomatic infection compared with T1 group (OR = 0.09, 95% CI, 0.02–0.35, *p* = 0.001); the T2 group of IgG had an 81% lower risk of developing a symptomatic infection compared to T1 group (OR = 0.19, 95% CI, 0.04–0.82, *p* = 0.026) (Figure 2).

In addition, lymphocytes are generally considered to be related to the severity of SARS-CoV-2 infection and have important clinical significance. Therefore, Model 3 is further adjusted by the effect of lymphocytes based on Model 2. In Model 3, T3 level, a specific quantification of IgG (≥3.44 S/CO) was associated with a 91% reduction in danger of developing a symptomatic infection in the fully adjusted models (IgG T2, OR = 0.19, 95% CI, 0.04–0.83, *p* = 0.028; IgG T3, OR = 0.09, 95% CI, 0.02–0.36, *p* = 0.001; Figure 2). As a result, multivariate regression showed that the highest tertile of IgG was a meaningfully independent and protective predictor of symptomatic infection after addressing the covariates in Model 2 and Model 3 (Figure 2).

### 3.3. Overall time to negative conversion

**Figure 3:**
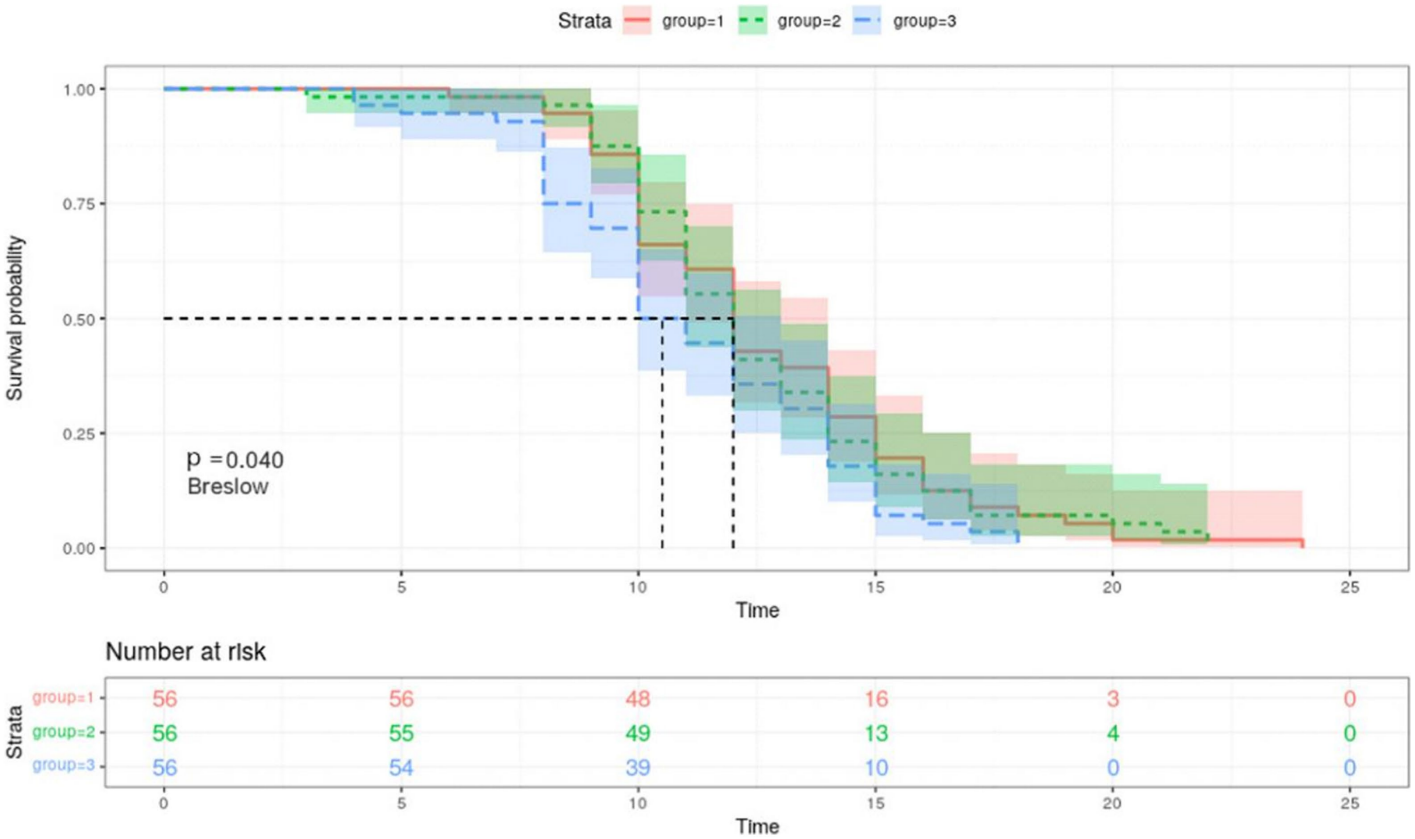
Kaplan–Meier survival curves for negative conversion of COVID 19 by tertiles of IgG. Group 1, the first tertile of IgG (lowest); Group 2, the second tertile of IgG; Group 3, the third tertile of IgG (highest). Kaplan–Meier curves show the time to negative conversion in patients with high, medium, or low level of IgG tertiles.

According to the Kaplan–Meier curves, the average time to negative conversion of patients with the lowest, medium, and highest tertile of IgG was 12.8, 12.6, and 11.2 days, respectively, and the median time of that was 12, 12, and 10 days, respectively. Generally, patients in the T3 group had shorter turnaround times than those at the T1 and T2 levels (Breslow *p* = 0.040, Figure 3). Nonetheless, in the multivariate Cox proportional hazards model (Table 3), IgG level was not significantly associated with overall time to negative conversion.

**Table 3:** Cox proportional hazards models of negative conversion time.

Variables		All patients (*N* = 168)	Model 1		Model 2		Model 3	
			Univariable HR (95%CI)	*P*	Multivariable HR(95%CI)	*P*	Multivariable HR(95%CI)	*P*
Gender	Female	79 (47.0)	1.00(Ref.)					
	Male	89 (53.0)	0.94 (0.69–1.27)	0.681	NA	NA	NA	NA
Age group, y	0-6	78 (46.4)	1.00(Ref.)					
	>6, ≤12	61 (36.3)	1.06 (0.76–1.48)	0.738	NA	NA	NA	NA
	>12, <18	29 (17.3)	0.70 (0.45–1.09)	0.117	NA	NA	NA	NA
Vaccination^b1^	Unvaccinated	50 (31.2)	1.00(Ref.)					
	Vaccinated	110 (68.8)	1.06 (0.76–1.48)	0.735	NA	NA	NA	NA
Tertiles of IgG	T1(0.01–0.42 S/CO)	56 (33.3)	1.00(Ref.)					
	T2(0.43–3.43 S/CO)	56 (33.3)	1.04 (0.72–1.51)	0.840	NA	NA	NA	NA
	T3(3.44–134.32 S/CO)	56 (33.4)	1.46 (1.00–2.12)	0.050	NA	NA	NA	NA
CRP levels	No.	165						
	≤10 mg/L	151 (91.5)	1.00(Ref.)					
	11–19 mg/L	7 (4.2)	0.60 (0.28–1.28)	0.186	NA	NA	NA	NA
	≥20 mg/L	7 (4.2)	1.27 (0.593–2.72)	0.539	NA	NA	NA	NA
PCT levels	No.	155						
	<0.1 ng/ml	105 (67.7)	1.00(Ref.)					
	0.1–0.25 ng/ml	30 (19.4)	0.61 (0.14–2.76)	0.524	NA	NA	NA	NA
	>0.25 ng/ml	20 (12.9)	0.92 (0.20–4.14)	0.912	NA	NA	NA	NA
SAA levels	No.	156						
	≤10 μg/ml	2 (1.3)	1.00(Ref.)					
	>10 μg/ml	154 (98.7)	0.51 (0.12–2.08)	0.350	NA	NA	NA	NA
IL-6 levels	No.	156						
	≤7 pg./ml	121 (77.6)	1.00(Ref.)					
	>7 pg./ml	35 (22.4)	1.04 (0.71–1.51)	0.854	NA	NA	NA	NA
ALT levels	No.	167						
	≤40 U/L	149 (89.2)	1.00(Ref.)					
	41-59 U/L	11 (6.6)	0.86 (0.46–1.58)	0.621	NA	NA	NA	NA
	≥60 U/L	7 (4.2)	0.77 (0.36–1.64)	0.493	NA	NA	NA	NA
AST levels	No.	168						
	≤40 U/L	132 (78.6)	1.00(Ref.)					
	41–59 U/L	21 (12.5)	0.87 (0.55–1.39)	0.564	NA	NA	NA	NA
	≥60 U/L	15 (8.9)	0.84 (0.49–1.43)	0.515	NA	NA	NA	NA
DDi levels	No.	158						
	≤0.50 μg/ml	121 (76.6)	1.00(Ref.)					
	0.51–0.99 μg/ml	26 (16.5)	1.08 (0.71–1.66)	0.716	NA	NA	NA	NA
	≥1.00 μg/ml	11 (7.0)	1.05 (0.57–1.96)	0.873	NA	NA	NA	NA
WBC	No.	167						
	Mean (SD), 10^9/L	6.1 (2.4)	1.00 (0.94–1.06)	0.955	NA	NA	NA	NA
NEUT	No.	167						
	Mean (SD), 10^9/L	3.0 (1.7)	1.00 (0.91–1.10)	0.987	NA	NA	NA	NA
LYM	No.	167						
	Mean (SD), 10^9/L	2.4 (1.8)	0.99 (0.92–1.07)	0.785	NA	NA	NA	NA
Hb	No.	167						
	Mean (SD), g/L	131.1 (11.5)	1.00 (0.99–1.01)	0.969	NA	NA	NA	NA
PLT	No.	167						
	Mean (SD),10^9/L	238.5 (70.7)	1.001 (0.999–1.004)	0.177	NA	NA	NA	NA
FIB	No.	163						
	Mean (SD), g/L	2.3 (0.6)	0.92 (0.70–1.21)	0.547	NA	NA	NA	NA
N-CT	No.	155						
	Mean (SD)	27.6 (4.8)	1.05 (1.02–1.08)	0.004	NA	NA	NA	NA
ORF1ab-CT	No.	151						
	Mean (SD)	27.3 (5.4)	1.04 (1.01–1.07)	0.007	1.04 (1.01–1.07)	0.007	1.04 (1.01–1.07)	0.014
CK	No.	166						
	Mean (SD), U/L	98.0 (41.8)	0.998 (0.994–1.002)	0.367	NA	NA	NA	NA
CK-MB	No.	166						
	Mean (SD), U/L	19.6 (8.9)	0.999 (0.980–1.019)	0.940	NA	NA	NA	NA
LDH	No.	167						
	Mean (SD), U/L	258.0 (67.5)	0.999 (0.997–1.002)	0.666	NA	NA	NA	NA
SARS-CoV-2 IgM	No.	168						
	Median (IQR), S/CO	0.14 (0.07, 0.30)	1.32 (1.01–1.73)	0.042	NA	NA	NA	NA

Model 2, the multivariate regression of IgG tertiles and time to negative conversion adjusted for age, gender, N-CT, ORF1ab-CT, SARS-CoV-2 IgM, and PLT. Model 3, multivariate regression of IgG tertiles and time to negative conversion adjusted for age, gender, N-CT, ORF1ab-CT, PLT, SARS-CoV-2 IgM, PLT, vaccination, and LYM. b1, the vaccine status of the 8 patients was missing. Missing data were not included in both univariate and multivariate regression.

## 4. Discussion

This study aimed to determine the relationship between IgG antibodies to wild-type SARS-CoV-2 antigens and children with symptomatic infection of the pandemic and duration to negative conversion. To our knowledge, this is the first clinical study to estimate antibody levels in children and adolescents grouped by tertiles in China so far.

As is known to us, IgG is usually produced 7 days after infection or vaccination, and we found that participants with high IgG levels were less likely to become symptomatic, had a shorter turnaround time, and had higher values of IgM and nucleic acid CT, which benefited from the vaccination, consistent with previous studies (14, 23). Since we measured serum anti-SARS-CoV-2 IgG levels during the acute phase, we considered the high IgG levels to be a consequence of vaccination. More importantly, it has been demonstrated that SARS-CoV-2 IgG antibodies are positively correlated with neutralizing antibody levels in a study, and the detection methods and reagents we used are consistent with this study (19). Then we performed a tertile analysis of SARS-CoV-2 IgG antibodies and revealed the association of serum SARS-CoV-2 IgG measurements with symptomatic infection in children. On the one hand, the Omicron variant can cause a faster immune response in children with high IgG levels, which can effectively inhibit virus replication (23). However, the IgG antibodies produced by the vaccine will decrease over time. Our results also support the necessity of boosters, which is why the Chinese government is actively promoting boosters. On the other hand, high IgG levels can effectively reduce the probability of symptomatic infection in children and promote a virus-negative conversion (14, 24). Fever, gastrointestinal symptoms, AST, and LDH are related to the severity of the disease (25, 26). There were a lower proportion of fever and anorexia and lower levels of AST and LDH among our patients with high tertiles of IgG due to the protective effect of antibodies after vaccination.

In this retrospective study, the risk of symptomatic infection with Omicron was significantly reduced among those with high IgG levels compared with low IgG levels, both meaningful in the univariate and multivariate regression model, which is consistent with previous studies (12, 13, 27). Although there is a high correlation between vaccine-induced neutralizing antibody levels and vaccine protection, previous studies have not demonstrated the exact number of neutralizing antibodies required to prevent SARS-Cov-2 infection in humans (18). Different from previous studies, we found that a specific quantification of IgG (≥3.44 S/CO) was associated with a 91% reduction in danger of developing a symptomatic infection in the fully adjusted models (OR = 0.09, 95% CI, 0.02-0.36, *p* = 0.001), which is beneficial to evaluate the need for supplemental vaccination based on quantitative antibody detection and optimize the vaccination strategy.

In terms of predicting the time to negative conversion, this is the first time to evaluate its association with IgG levels by tertiles. Through KM curve analysis, we found that patients with a high IgG level had a shorter time to negative conversion in favor of reducing medical burden. However, in the COX multivariate analysis model, there is no correlation between the IgG level and the time to negative conversion, possibly due to insufficient sample size, and in-depth research is in need in the future.

The strength of this study, which is currently the first cohort study to assess levels of IgG antibodies to wild-type SARS-CoV-2 antigens in children and adolescents with Omicron symptomatic infection, includes detailed demographic information, clinical symptoms, and laboratory findings. More importantly, we found a quantitative level of the significant protective effect of IgG by tertile grouping, that is, IgG ≥3.44S/CO. Briefly, measures of IgG were beneficial to predicting those at risk of symptomatic infection and distinguishing a quantitative level (IgG ≥3.44 S/CO) to assess vaccine efficacy and optimize the vaccination strategy.

This study is limited by its observational design and limited sample size and we cannot rule out residual confounders, especially unmeasured variables. At the same time, we cannot determine when IgG falls below 3.44 S/CO after vaccination. Another factor that will limit the applicability of this study is the ongoing evolution of SARS-CoV-2. Unfortunately, the determination of any IgG level correlating with immunity will inevitably change with subsequent variants. The Omicron variant is associated with the potential for evading accurate diagnostics and less severe COVID-19 symptoms (28, 29). However, we did not evaluate those potential patients for evaded accurate diagnostics with limited detection. We also could not determine the association between the tertiles of IgG levels and severe symptoms based on the limited data. Furthermore, the current Omicron lineages BQ.1.1 and XBB.1, for example, may be much more likely to cause symptomatic infection at IgG levels that would have been protective against earlier variants, which limits any serologic approach to booster strategies (30). Last but not least, our detection method could not make a differentiation in the IgG isotype present, which may be a hot topic in the future. More research is in need in the future, such as exploring associations between IgG levels and outcomes in different age groups, investigating the long-term persistence of IgG antibodies, and assessing the effectiveness of booster strategies based on IgG antibody levels.

## 5. Conclusion

We demonstrate that level of IgG antibodies to wild-type SARS-CoV-2 antigens is an independent predictor of symptomatic infection. High IgG levels can inhibit viral replication and vastly reduce the risk of developing a symptomatic infection, especially when IgG quantitative detection was ≥3.44 S/CO, a potential threshold for protection and booster strategy. Children with high IgG levels required a shorter time to negative conversion, although the relationship is not robust. More data and research are needed in the future to validate the predictive models.

## Data availability statement

The original contributions presented in the study are included in the article/supplementary material, further inquiries can be directed to the corresponding authors.

## Ethics statement

This study has been reviewed and approved by the Ethics Committee of Guangdong Provincial Hospital of Chinese Medicine (Ethic Review No. ZF2022-246-01). It complies with the principles of Helsinki Declaration and medical ethics. The studies were conducted in accordance with the local legislation and institutional requirements. Written informed consent for participation in this study was provided by the participants’ legal guardians/next of kin.

## Author contributions

JY, JG, and BY had full access to all the data in the study and take responsibility for the integrity of the data and the accuracy of the data analysis. JY, LD, XC, JH, YL, BY, and JG conceived and designed the study. JY, HW, XC, JH, TH, and LD performed the acquisition, analysis, or interpretation of data. XC, JH, HW, and AO conducted the statistical analysis and drew figures. JY, LD, XC, and JG drafted the manuscript. JY, HW, BY, and JG took responsibility for administrative, technical, and material support. XC and JH carried out additional work in response to comments from reviewers, including re-reviewing and analyzing data, drawing figures, and critically revising and reviewing the article. All authors critically reviewed the manuscript for important intellectual content, approved the final manuscript as submitted and agree to be accountable for all aspects of the work.

## Funding

This study was supported by National Key R&D Programmes (NKPs) of China, aimed at Evidence-based evaluation of Fuzheng Jiedu Fang to block severe transformation of infection with SARS-Cov-2 Delta variant (No.2022YFC0867400). It was also supported by State Administration of Traditional Chinese Medicine, an urgent project for Chinese Medicine on Novel Coronavirus Pneumonia (Nos. 2022ZYLCYJ07-3 and 2022ZYLCYJ11-2), Guangdong Provincial Administration of Traditional Chinese Medicine (No. 2022ZYYJ04), State Key Laboratory of Dampness Syndrome of Chinese Medicine in the Provincial Cooperation Administration (No. SZ2022XG01), and Xiaorong Luo’s Renowned Expert Inheritance Studio (No. 14GG2X02). The sponsors had no role in the design and conduct of the study; collection, management, analysis, and interpretation of the data; preparation, review, or approval of the manuscript; and decision to submit the manuscript for publication.

## Conflict of interest

The authors declare that the research was conducted in the absence of any commercial or financial relationships that could be construed as a potential conflict of interest.

## Publisher’s note

All claims expressed in this article are solely those of the authors and do not necessarily represent those of their affiliated organizations, or those of the publisher, the editors and the reviewers. Any product that may be evaluated in this article, or claim that may be made by its manufacturer, is not guaranteed or endorsed by the publisher.

## Author disclaimer

The contents are solely the responsibility of the authors and do not necessarily represent the official views of the State.

## References

[1] Karim SSA Karim QA. Omicron SARS-cov-2 variant: a new chapter in the covid-19 pandemic. Lancet. (2021) 398:2126–8. doi: 10.1016/S0140-6736(21)02758-6, PMID: 34871545 PMC8640673

[2] Chen J Wei GW. Omicron Ba.2 (b.1.1.529.2): high potential for becoming the next dominant variant. J Phys Chem Lett. (2022) 13:3840–9. doi: 10.1021/acs.jpclett.2c00469, PMID: 35467344 PMC9063109

[3] Cheng V Ip JD Chu A Tam AR Chan WM Abdullah S. Rapid spread of severe acute respiratory syndrome coronavirus 2 (SARS-cov-2) omicron subvariant Ba.2 in a single-source community outbreak. Clin Infect Dis. (2022) 75:e44–9. doi: 10.1093/cid/ciac203, PMID: 35271728 PMC8992238

[4] Chen Z Deng X Fang L Sun K Wu Y Che T. Epidemiological characteristics and transmission dynamics of the outbreak caused by the SARS-CoV-2 Omicron variant in Shanghai, China: a descriptive study. Lancet Reg Health West Pac. (2022) 29:100592. doi: 10.1016/j.lanwpc.2022.100592, PMID: 36090701 PMC9448412

[5] Fleming-Dutra KE Britton A Shang N Derado G Link-Gelles R Accorsi EK. Association of prior bnt162b2 covid-19 vaccination with symptomatic SARS-cov-2 infection in children and adolescents during omicron predominance. JAMA. (2022) 327:2210–9. doi: 10.1001/jama.2022.7493, PMID: 35560036 PMC9107063

[6] Wang L Berger NA Kaelber DC Davis PB Volkow ND Xu R. Incidence rates and clinical outcomes of SARS-cov-2 infection with the omicron and delta variants in children younger than 5 years in the us. JAMA Pediatr. (2022) 176:811–3. doi: 10.1001/jamapediatrics.2022.0945, PMID: 35363246 PMC8976262

[7] Xia S Wang L Zhu Y Lu L Jiang S. Origin, virological features, immune evasion and intervention of SARS-cov-2 omicron sublineages. Signal Transduct Target Ther. (2022) 7:241. doi: 10.1038/s41392-022-01105-9 35853878 PMC9295084

[8] Tian D Sun Y Xu H Ye Q. The emergence and epidemic characteristics of the highly mutated SARS-cov-2 Omicron variant. J Med Virol. (2022) 94:2376–83. doi: 10.1002/jmv.27643, PMID: 35118687 PMC9015498

[9] Butt AA Dargham SR Coyle P Yassine HM Al-Khal A Abou-Samra AB. COVID-19 disease severity in persons infected with omicron Ba.1 and Ba.2 sublineages and association with vaccination status. Jama Intern Med. (2022) 182:1097–99. doi: 10.1001/jamainternmed.2022.3351, PMID: 35994264 PMC9396464

[10] Muhsen K Maimon N Mizrahi AY Boltyansky B Bodenheimer O Diamant ZH. Association of receipt of the fourth bnt162b2 dose with omicron infection and covid-19 hospitalizations among residents of long-term care facilities. JAMA Intern Med. (2022) 182:859–67. doi: 10.1001/jamainternmed.2022.2658, PMID: 35737368 PMC9227688

[11] Barouch DH. COVID-19 vaccines - immunity, variants, boosters. N Engl J Med. (2022) 387:1011–20. doi: 10.1056/NEJMra2206573, PMID: 36044620 PMC9454645

[12] Earle KA Ambrosino DM Fiore-Gartland A Goldblatt D Gilbert PB Siber GR. Evidence for antibody as a protective correlate for covid-19 vaccines. Vaccine. (2021) 39:4423–8. doi: 10.1016/j.vaccine.2021.05.063, PMID: 34210573 PMC8142841

[13] Gilbert PB Montefiori DC McDermott AB Fong Y Benkeser D Deng W. Immune correlates analysis of the mrna-1273 covid-19 vaccine efficacy clinical trial. Science. (2022) 375:43–50. doi: 10.1126/science.abm3425, PMID: 34812653 PMC9017870

[14] Asamoah-Boaheng M Goldfarb DM Karim ME O'Brien SF Wall N Drews SJ. The relationship between anti-spike SARS-cov-2 antibody levels and risk of breakthrough COVID-19 among fully vaccinated adults. J Infect Dis. (2023) 227:339–43. doi: 10.1093/infdis/jiac403 36197948 PMC9619727

[15] Xue JH Wang YJ Li W Li QL Xu QY Niu JJ. Anti-receptor-binding domain immunoglobulin G antibody as a predictor of seropositivity for anti-SARS-CoV-2 neutralizing antibody. Arch Pathol Lab Med. (2022) 146:814–21. doi: 10.5858/arpa.2022-0041-SA, PMID: 35380612

[16] Long QX Tang XJ Shi QL Li Q Deng HJ Yuan J. Clinical and immunological assessment of asymptomatic SARS-cov-2 infections. Nat Med. (2020) 26:1200–4. doi: 10.1038/s41591-020-0965-6, PMID: 32555424

[17] Long QX Liu BZ Deng HJ Wu GC Deng K Chen YK. Antibody responses to SARS-cov-2 in patients with covid-19. Nat Med. (2020) 26:845–8. doi: 10.1038/s41591-020-0897-1, PMID: 32350462

[18] Wang Y. Standardised neutralising antibody assays are needed for evaluating covid-19 vaccines. EBioMedicine. (2021) 73:103677. doi: 10.1016/j.ebiom.2021.103677, PMID: 34742128 PMC8564504

[19] Cheng ZA-O Huang H Zheng P Xue M Ma J Zhan Z. Humoral immune response of BBIBP COVID-19 vaccination before and after the booster immunization. Allergy. (2022) 17:2404–14. doi: 10.1111/all.15271, PMID: 35255171 PMC9111230

[20] Tabata S Imai K Kawano S Ikeda M Kodama T Miyoshi K. Clinical characteristics of COVID-19 in 104 people with SARS-CoV-2 infection on the diamond princess cruise ship: a retrospective analysis. Lancet Infect Dis. (2020) 20:1043–50. doi: 10.1016/S1473-3099(20)30482-5, PMID: 32539988 PMC7292609

[21] Stone GW Maehara A Lansky AJ de Bruyne B Cristea E Mintz GS. A prospective natural-history study of coronary atherosclerosis. N Engl J Med. (2011) 364:226–35. doi: 10.1056/NEJMoa1002358, PMID: 21247313

[22] Kang SJ Cho YR Park GM Ahn JM Han SB Lee JY. Predictors for functionally significant in-stent restenosis: an integrated analysis using coronary angiography, IVUS, and myocardial perfusion imaging. JACC Cardiovasc Imaging. (2013) 6:1183–90. doi: 10.1016/j.jcmg.2013.09.006, PMID: 24229771

[23] Li X Wu L Qu Y Cao M Feng J Huang H. Clinical characteristics and vaccine effectiveness against SARS-CoV-2 omicron subvariant BA.2 in the children. Signal Transduct Target Ther. (2022) 7:203. doi: 10.1038/s41392-022-01023-w 35764610 PMC9240082

[24] Chen H Qin R Huang Z He L Luo W Zheng P. Characteristics of covid-19 patients based on the results of nucleic acid and specific antibodies and the clinical relevance of antibody levels. Front Mol Biosci. (2020) 7:605862. doi: 10.3389/fmolb.2020.605862, PMID: 33585558 PMC7874027

[25] Zhang JJY Lee KS Ang LW Leo YS Young BE. Risk factors for severe disease and efficacy of treatment in patients infected with COVID-19: a systematic review, Meta-analysis, and Meta-regression analysis. Clin Infect Dis. (2020) 71:2199–206. doi: 10.1093/cid/ciaa576, PMID: 32407459 PMC7239203

[26] Mao R Qiu Y He JS Tan JY Li XH Liang J. Manifestations and prognosis of gastrointestinal and liver involvement in patients with COVID-19: a systematic review and meta-analysis. Lancet Gastroenterol Hepatol. (2020) 5:667–78. doi: 10.1016/S2468-1253(20)30126-63 32405603 PMC7217643

[27] Khoury DS Cromer D Reynaldi A Schlub TE Wheatley AK Juno JA. Neutralizing antibody levels are highly predictive of immune protection from symptomatic SARS-cov-2 infection. Nat Med. (2021) 27:1205–11. doi: 10.1038/s41591-021-01377-8, PMID: 34002089

[28] Sharma D Notarte KI Fernandez RA Lippi G Gromiha MM Henry BM. In silico evaluation of the impact of omicron variant of concern sublineage BA.4 and BA.5 on the sensitivity of RT-qPCR assays for SARS-CoV-2 detection using whole genome sequencing. J Med Virol. (2023) 95:e28241. doi: 10.1002/jmv.28241, PMID: 36263448 PMC9874926

[29] Fernández-de-Las-Peñas C Notarte KI Peligro PJ Velasco JV Ocampo MJ Henry BM. Long-COVID symptoms in individuals infected with different SARS-CoV-2 variants of concern: a systematic review of the literature. Viruses. (2022) 14:2629. doi: 10.3390/v14122629, PMID: 36560633 PMC9785120

[30] Uraki R Ito M Furusawa Y Yamayoshi S Iwatsuki-Horimoto K Adachi E. Humoral immune evasion of the omicron subvariants BQ.1.1 and XBB. Lancet Infect Dis. (2023) 23:30–2. doi: 10.1016/S1473-3099(22)00816-7, PMID: 36495917 PMC9729000

